# Nanoemulsion-Based Delivery Systems to Improve Functionality of Lipophilic Components

**DOI:** 10.3389/fnut.2014.00024

**Published:** 2014-12-05

**Authors:** Isabel Odriozola-Serrano, Gemma Oms-Oliu, Olga Martín-Belloso

**Affiliations:** ^1^Department of Food Technology, University of Lleida, Lleida, Spain

**Keywords:** nanoemulsions, lipophilic nanoparticles, functionality, essential oils, antimicrobials, bioactive compounds

## Abstract

The use of active lipophilic substances such as antimicrobials and health-related compounds in the food industry is still a challenge due to their poor water solubility and instability in food formulations. Nano-sized structures such as nanoemulsions of oil-in-water are regarded as useful tools with a great potential in the food sector to incorporate food ingredients. Reducing the size of the active compounds incorporated within a solution would increase the surface area per mass unit of nanoemulsions, thus enhancing solubility and stability in foods. In addition, the ability of the active lipids to penetrate across biological membranes is also enhanced, thus boosting their biological functionality. An overview of the most significant studies reporting data about the potential benefits of active lipid nanoemulsions over conventional emulsions is presented.

## Introduction

Nowadays, consumers are demanding safe and healthy food products being at the same time as natural as possible. Therefore, new trends in the food market are leading toward a more rational use of food ingredients, such as preservatives, minimizing the concentration of synthetic additives, or even replacing them with natural substances. Nanotechnology is bursting into the food sector with several applications since it offers new insights to develop safer and healthier foodstuffs. The use of nanoemulsion-based delivery systems, as a method to encapsulate active lipophilic ingredients dispersed in aqueous media, is emerging as a potential tool to design new food products. Nanoemulsions are defined as oil droplets, with particle sizes comprised between 10 and 100 nm, dispersed in aqueous media (1). These systems have been thought to have several advantages over conventional emulsions as colloidal delivery systems due to their smaller particle size. Nanoemulsions seem to present optical transparency, enhanced functionality, and physical stability, which would make them very attractive for food products. The aim of this review article was to provide an insight into the use of nanoemulsions containing active lipophilic as a strategy to improve their functionality.

## Nanoemulsion Formation

The formulation of nanoemulsions consists on mixing at least three components being oil and aqueous phases plus a stabilizer. The lipid phase of oil-in-water nanoemulsions generally acts as a carrier of lipophilic active compounds and is the dispersed phase in the continuous phase. Usually, the lipophilic active ingredients are solubilized in the oil phase prior to the formation of emulsions. The oil phase can be formulated with different non-polar compounds, such as triglycerides, mineral oils, or essential oils. On the other hand, the aqueous phase of food emulsions might contain a variety of water-soluble constituents, including minerals, acids, bases, flavors, preservatives, vitamins, sugars, surfactants, proteins, or polysaccharides (2). Besides the lipid and aqueous phases, the formulation of nanoemulsions requires the use of stabilizers such as emulsifiers and hydrocolloids to prevent the breakdown of the nanoemulsion structure once it is formed. Emulsifiers are surface-active amphiphilic molecules; thus, the lipophilic part has affinity for non-polar media and the hydrophilic part has affinity for polar media. They are able to adsorb at the oil–water interface of droplet surfaces during emulsification, thus protecting droplets against re-coalescence or aggregation (3). Hydrocolloids have been extensively used in food formulations for their thickening properties when incorporated into aqueous phase. Modifying the rheology of the aqueous phase not only changes the emulsions texture and mouth-feel but also minimizes the droplets movement in the fluid so retarding gravitational separation (creaming or sedimentation) of lipid particles (4). In general, lipid nanoparticles can be prepared using two different approaches: high-energy or low-energy devices. High-energy methods consist in applying high disruptive forces with mechanical devices, capable of causing the breakup of oil droplets and disperse them into the water phase. Low-energy approaches rely on the spontaneous formation of tiny oil droplets within mixed oil–water–emulsifier systems when the solution or environmental conditions are altered, such as composition or temperature (5).

## Functionality of Lipid Nanoparticles

There are several lipophilic active ingredients used in food industry such as certain antimicrobials or bioactive compounds that present low water solubility and instability when incorporating them in food formulations with high water content.

### Antimicrobials

The use of natural essential oils as antimicrobials is becoming more and more popular due to the consumers demand of food free from synthetic additives. Essential oils contain a complex mixture of non-volatile and volatile compounds produced by aromatic plants as secondary metabolites. In particular, the antimicrobial action of essential oils has been attributed to their phenolic compounds and their interaction with microbial cell membranes, causing the leakage of cytoplasmatic constituents and, therefore, the loss of cell viability (6). Moreover, other mechanisms may be involved in microbial inactivation by essential oils since they could interfere with membrane function, altering its electron transport, nutrient uptake, proteins, nucleic acid synthesis, and enzyme activity (7). However, the use of essential oils in foods still presents several limitations considering their low solubility in aqueous media, as well as their toxicological, organoleptical, and economical aspects when they are incorporated in high doses (6). Several recent research works report the formation of nanoemulsions containing essential oils as a strategy to improve their functionality (Table 1). Essential oils incorporated in nanoemulsions seem to penetrate faster in the microbial membranes due to the increased area per weight unit (8–11). This would allow reducing the concentration to achieve an equivalent or even greater bacterial effect over conventional emulsions. However, processing method to obtain essential oil nanoemulsions determine their antimicrobial activity. Salvia-Trujillo et al. (11) reported that ultrasound processing diminished the antimicrobial potential of lemongrass oil–alginate nanoemulsions against *Escherichia coli* in comparison with microfluidized nanoemulsions.

**Table 1:** **Functionality of nanoemulsions containing natural essential oils**.

Antimicrobial compounds	Concentration	Lipid carrier	Stabilizers	Functionality	Reference
Thyme essential oil	250 mL/kg of antimicrobials in the lipid carrier	Corn oil	Tween 80 Sodium dodecyl sulfate Lauric arginate	The combination of thyme oil and antimicrobial surfactant had an antagonist impact on the overall antimicrobial efficacy	Ziani et al. (8)
d-limonene and other terpenes	50 mg/kg of antimicrobials in the nanoemulsions	Sunflower and palm oil	Soy lecithin Tween 20 Glycerol monooleate Modified starch	Low concentrations of the nanoencapsulated terpenes delayed the microbial growth or completely inactivated the microorganisms without altering the organoleptic properties of fruit juices	Donsi et al. (9)
Peppermint essential oil	200 mL/kg of antimicrobials in the lipid carrier	Medium chain triglyceride	Modified starch	Nanoemulsions showed prolonged antibacterial activity in comparison with non-encapsulated oil	Liang et al. (10)
Lemongrass essential oil	100 mL/kg of antimicrobials in the lipid carrier	–	Tween 20	Nanoemulsions produced by microfluidization showed a faster inactivation kinetics against *E. coli* in comparison with sonicated nanoemulsions	Salvia-Trujillo et al. (11)

### Bioactive compounds

There are many lipophilic bioactive compounds that are susceptible to be incorporated in foods due to their health promoting properties. Carotenoids, omega-3 fatty acids, polyphenols, flavonoids, phytosterols, and tocopherols are the main lipophilic bioactive compounds used to fortify foods. The incorporation of highly hydrophobic bioactive compounds in food is a challenge not only due to their poor water solubility, fast oxidation, and low sensorial detection thresholds but also because of their low bioaccessibility after the digestion in the gastrointestinal tract. The size and composition of the droplets in nanoemulsions influences the rate and extent of lipid digestion, as well as the bioavailability of lipophilic active compounds. The rate of lipid digestion increases as the droplet size decreases, which has been attributed to the increase in surface area of lipid exposed to intestinal juices containing lipase (12, 13). In addition, several authors (14, 15) reported that the smaller the droplet size of nanoemulsions, the higher the bioaccessibility of bioactive compounds encapsulated. It is known that not all lipids and fats present the same behavior through the gastrointestinal tract, thus the type of oil used in the formation of nanoemulsions will directly influence their biological activity. In this sense, it has been reported that the *in vitro* digestion of nanoemulsions containing lipid carriers with a longer chain of fatty acids led to a higher bioaccesibility of lipophilic active compounds (16–18). The emulsifier type and concentration will impact the susceptibility of the lipid droplets to coalescence and break up within the gastrointestinal tract, thereby altering the total surface area of lipid exposed to lipase action and consequently the rate of lipid digestion and bioaccessibility (19, 20). The most significant research studies focused on describe the benefits of incorporating bioactive compounds in nanoemulsions are summarized in Table 2.

**Table 2:** **Functionality of nanoemulsions containing bioactive compounds**.

Bioactive compounds	Concentration^a^	Lipid carrier	Stabilizers	Functionality	Reference
β-carotene	5 mg/kg	Corn oil, medium chain triacylglycerols, or orange oil	Tween 20	β-carotene bioaccessibility decreased in the following order: corn oil > medium chain triacylglycerols > orange oil	Qian et al. (17)
β-carotene	3 mg/kg	Medium chain triglyceride	Modified starch	β-carotene bioaccesibility improved significantly after encapsulation in nanoemulsions	Liang et al. (15)
β-carotene	5 mg/kg	Corn oil and Miglyol 812	Tween 20	In low fat nanoemulsions (1%), the bioaccessibility of β-carotene increase with increasing the fatty acids chain length	Salvia-Trujillo et al. (14)
β-carotene	5 mg/kg	Corn oil	Tween 20	β-carotene bioaccesibility decreased as the initial droplet size increased	Salvia-Trujillo et al. (18)
Vitamin E	8 mg/kg	Medium chain triglyceride	Tween 80	Bioaccessibility of vitamin E encapsulated in nanoemulsions was higher compared with conventional emulsions	Mayer et al. (20)
Coenzyme Q10 Heptadecanoic acid	1 mg/kg	Corn oil, Mineral oil	Tween 80	Bioavailability of coenzyme Q10 and heptadecanoic acid was the highest when they were encapsulated in droplets with the smallest size	Cho et al. (13)
Curcumin	1.5 mg/kg	Long, medium, and short chain triacylglycerols	β-lactoglobulin	The higher the chain length of fatty acids, the higher the bioaccessibility of curcumin	Ahmed et al. (16)
Curcumin	1 mg/kg	Corn oil	Tween 20 or Sodium dodecyl sulfate	Curcumin exhibited high bioavailability in the presence of Tween 20 in comparison nanoemulsions with dodecyltrimethylammonium bromide	Pinheiro et al. (19)

*^a^Concentration of the bioactive compound in the lipid carrier*.

## Future Prospects

Nanoemulsions constitute one of the most promising systems to improve solubility and functionality of lipophilic active food ingredients. However, the promising expectation arising from the recent publication data are based on few research papers. Therefore, there is a need for further studies including a wide range of active compounds loaded in nanoemulsions to elucidate the real benefits of nanoemulsions depending on the kind of lipid nanoparticle. Despite lipid nanoparticles show similar digestibility pattern compared to conventionally emulsions, their toxicological safety cannot be certainly assured. The biological path of lipid nanoparticles once they entry in human gut should be described to assess tissue location and possible toxicity.

## Conflict of Interest Statement

The authors declare that the research was conducted in the absence of any commercial or financial relationships that could be construed as a potential conflict of interest.
